# Impact of falls on early mortality from severe traumatic brain injury

**DOI:** 10.1186/1752-2897-3-9

**Published:** 2009-07-30

**Authors:** Linda M Gerber, Quanhong Ni, Roger Härtl, Jamshid Ghajar

**Affiliations:** 1 Department of Public Health, Weill Cornell Medical College, New York, USA; 2 Department of Medicine, Weill Cornell Medical College, New York, USA; 3 Department of Neurological Surgery, Weill Cornell Medical College, New York, USA; 4 Brain Trauma Foundation, New York, USA

## Abstract

**Background:**

The causes of severe traumatic brain injury (TBI) vary by age and other demographic characteristics. Mortality after trauma is higher for elderly than younger patients. This study is based on 2779 patients with severe TBI treated at 24 trauma centers enrolled in a New York State quality improvement program. The prospectively collected database includes information on age, sex, mechanism of injury, initial Glasgow Coma Scale score, blood pressure, pupillary assessment, and CT scan findings. This multi-center study was conducted to explore the impact of falls on early mortality from severe TBI among the elderly.

**Results:**

After exclusion criteria were applied, a total of 2162 patients were eligible for analysis. Falls contributed to 21% of all severe TBI, 12% occurring from > 3 meters and 9% from < 3 meters. Two-week mortality ranged from 18% due to injuries other than falls to 31% due to falls from < 3 meters (p =< 0.0001). Mortality after a severe TBI is much greater among older people, reaching 58% for people 65 years and older sustaining a fall from < 3 meters.

**Conclusion:**

Among those 65 and older, falls contributed to 61% of all injuries and resulted in especially high mortality among individuals experiencing low falls. Preventive efforts directed toward older people to avoid falls from < 3 meters could have a significant impact on mortality.

## Introduction

Traumatic brain injury (TBI) is the leading cause of death among ages 1 to 44 years. Each year in the United States there are 50,000 deaths from TBI and an additional 70,000 to 90,000 individuals are left with permanent neurological disabilities [1]. TBI is the leading cause of death among all trauma-related deaths [2,3].

Since 2000, a quality improvement (QI) program exists in New York for tracking the treatment of severe TBI patients (Glasgow Coma Scale [GCS] score < 9) in 24 of the 46 state designated trauma centers. The program, initiated by the Brain Trauma Foundation and funded through the New York State Department of Health Bureau of Emergency Medical Services, is designed to assess and implement adoption of the evidence-based *Guidelines for the Management of Severe Traumatic Brain Injury*. The Guidelines were formulated and disseminated in 1995 [4] and updated in 2000 [5] and 2006 [6] by the Brain Trauma Foundation in collaboration with the American Association of Neurological Surgeons.

Severe TBI results in prolonged hospital stays and is the most common cause of traumatic deaths [7,8]. Most severe TBIs are due to falls or motor vehicle-related incidents [9]. The populations at risk for the causes of injury vary by age and other demographic characteristics. As the proportion of elderly increases, falls occurring within this group that result in severe TBI, with patients often sustaining serious multisystem injuries [10], warrant greater attention.

This report describes the characteristics of severe TBI among New York State residents and highlights the impact of falls among the elderly. These data are important in not only informing the development of targeted injury prevention programs, but also to estimate the needs of the clinical facilities where these patients are most often treated.

## Methods

Data from the QI TBI Program's internet-based database, TBI-trac™, were used. The database includes information on mechanism of injury, patient age, sex, race, and seatbelt and helmet use. Data are also collected from the patient's stay in the intensive care unit on physiologic variables, the presence of secondary insults (hypotension, hypoxia and cerebral hypoperfusion) and any therapies to reduce elevated intracranial pressure (ICP). Outcome is assessed at two weeks.

Patients are eligible for data entry into the TBI-trac™ database if they present with a GCS score less than nine for at least six hours from the time of injury, after stabilization and resuscitation efforts. The mechanism of injury must be consistent with TBI (e.g. fall, assault, motor vehicle crash). Patients in coma as the result of non-traumatic events such as subarachnoid hemorrhage or stroke are not eligible for data collection. There are no age-based exclusion criteria. Patients who died prior to arrival to the trauma center or those who died or were declared brain dead in the emergency department are not included in data collection. Data collection stops when the patient achieves a GCS motor score of 6 or when the patient dies or is declared brain dead. All data are prospectively abstracted and entered into the database by the trauma program manager at each center. The following definitions apply to the database.

**Mechanism of injury- **defined by data abstracted from the ambulance prehospital care record.

**Hypotension**- systolic blood pressure less than 90 mmHg for individuals greater than or equal to 12 years, less than 80 mmHg for individuals between the ages of 6 to 11 years, less than 75 mmHg for individuals 1–5 years old, and less than 65 mmHg for individuals less than or equal to 1 year.

**Abnormal pupil status- **asymmetry or bilaterally fixed and dilated pupils.

**Abnormal CT scan**- basal cisterns not fully open or multiple parenchymal hemorrhages or shift of the ventricles or subarachnoid hemorrhage.

**Outcome GCS**- 14-day post-injury GCS score.

**Mortality**- 14-day post-injury assessment of the patient's condition as alive or dead.

The research protocol was approved or deemed exempt from review by the institutional review board at each participating trauma center. The database contains no patient identifiers, and confidentiality is maintained for each center's data.

### Statistical methods

The chi-square test was used to evaluate the association between severity of injury and mechanism of injury, as well as two-week outcome and mechanism of injury. Logistic regression analyses predicting falls were used to estimate the odds ratios, 95% confidence intervals, and p values of the predictor variables, controlling for sex, race, and year of injury. All statistical tests were two-sided and p < 0.05 was considered statistically significant. Analyses were performed in SAS version 9.1 (SAS Institute, Inc., Cary, North Carolina).

## Results

Data for 2779 patients were entered in the database from June 6, 2000 through December 31, 2008. Patients were excluded if they had a GCS score greater than or equal to nine on day one (136 patients), or a GCS motor score of six on any day (20 patients). These patients were excluded from the analysis because they do not meet the definition of severe TBI according to the Brain Trauma Foundation Guidelines. Patients were also excluded if they had a GCS score of three with pupils bilaterally fixed and dilated and not pharmacologically paralyzed (225 patients) or a daily or outcome GCS score equal to four with pupils bilaterally fixed and dilated or missing pupil information (118 patients). These patients were excluded because they would not be expected to benefit from acute care interventions. Patients with a recorded time to study hospital greater than 24 hours (42 patients), a transport time to study hospital less than 10 minutes (40 patients), or were missing mechanism of injury (12 patients) or outcome assessment (24 patients) were also excluded. After exclusion criteria were applied, a total of 2162 patients were eligible for analysis.

The characteristics of the study population are shown in Table 1. The majority of patients were male (75%) with a mean age of 36 years. Approximately 12% of patients were 65 or older. GCS scores on day 1 were less than 6 in 50% of patients, the remainder being between 6 and 8.

**Table 1:** Characteristics of the study population

	Total
	(N = 2162)
Age (yrs)	**N (%)**

0–39	1299 (60.1)

40–64	609 (28.2)

≥65	254 (11.7)

Male	1630 (75.4)

Race	

White	1627 (75.4)

Black	323 (15.0)

Other	208 (9.6)

Mechanism of Injury	

Fall: < 3 meters	202 (9.3)

Fall: > 3 meters	248 (11.5)

Other	1712 (79.2)

Day 1 GCS score	

3 – 5	1051 (50.1)

6 – 8	1046 (49.9)

Day 1 Hypotension	317 (14.5)

Day 1 Abnormal Pupil Status	440 (20.6)

Abnormal CT Scan	1671 (81.6)

Mortality	420 (19.4)

Outcome GCS	

3 – 8	557 (33.0)

9 – 15	1133 (67.0)

Table 2 presents mechanism of injury by age group. The leading causes of severe TBI were motor vehicle crashes (35%), followed by falls (21%), and pedestrians struck by a motor vehicle (16%), accounting for approximately 72% of all injuries. When examined by age group, approximately 3/4 (76%) of motor vehicle crashes occurred among those under 40 while almost half (47%) of falls from < 3 meters were among those 65 years and older.

**Table 2:** Mechanism of injury by age

Mechanism of Injury		Age		
		
	Total N (%)	0-39 N (%)	40-64 N (%)	65+ N (%)
Falls: < 3 meters	202 (9.3)	38 (2.9)	69 (11.3)	95 (37.4)

Falls: > 3 meters	248 (11.5)	87 (6.7)	102 (16.8)	59 (23.2)

Pedestrian struck by motor vehicle:	350 (16.2)	202 (15.6)	105 (17.3)	43 (16.9)

Motor Vehicle:	752 (34.8)	575 (44.3)	140 (23.0)	37 (14.6)

Assault:	138 (6.4)	82 (6.3)	53 (8.7)	3 (1.2)

Gunshot:	66 (3.1)	50 (3.9)	15 (2.5)	1 (0.4)

Motorcycle, Bicycle, Snowmobile:	283 (13.1)	184 (14.2)	94 (15.4)	5 (2.0)

Other:	80 (3.7)	49 (3.8)	23 (3.8)	8 (3.2)

Sports:	43 (2.0)	32 (2.5)	8 (1.3)	3 (1.2)

Total:	2162	1229	609	254

Age was an independent predictor of falls from < 3 meters and from > 3 meters after controlling for sex, race, and year of injury (Table 3). Specifically, adults 65 years and older were 29.4 times more likely to have falls from < 3 meters than patients less than 40 years. Adults 40–64 were also at increased risk of falls from < 3 meters (odds ratio: 4.9 [95% CI: 3.3–7.4]). Increasing age also contributed significantly to falls from > 3 meters; adults aged 40–64 and those 65 and older were 3.2 and 8.6 times more likely, respectively, to have falls from > 3 meters compared with younger patients. Males had almost twice the risk of falls from > 3 meters compared with females (95% CI: 1.3 – 2.6).

**Table 3:** Logistic regression predicting falls

	Falls: < 3 meters*			
	Crude OR (95% CI)	P-value	Adjusted OR (95% CI)	P-value

Age				

0 – 39	1		1	

40 – 64	4.87 (3.23, 7.34)	<0.0001	4.91 (3.25, 7.41)	<0.0001

65+	29.35 (19.13, 45.03)	<0.0001	29.39 (19.02, 45.43)	<0.0001

Sex				

Female	1		1	

Male	0.73 (0.53, 1.00)	0.05	0.94 (0.66, 1.36)	0.75

Race				

White	1		1	

Black	0.80 (0.52, 1.24)	0.32	1.22 (0.75, 1.97)	0.42

Other	1.11 (0.68, 1.80)	0.68	1.12 (0.65, 1.93)	0.68

Year of Injury (every year increment)	0.96 (0.90, 1.02)	0.17	0.96 (0.89, 1.03)	0.21

	Falls: > 3 meters**			

	Crude OR (95% CI)	P-value	Adjusted OR (95% CI)	P-value

Age				

0 – 39	1		1	

40 – 64	3.14 (2.31, 4.27)	<0.0001	3.16 (2.32, 4.31)	<0.0001

65+	7.96 (5.40, 11.74)	<0.0001	8.62 (5.79, 12.83)	<0.0001

Sex				

Female	1		1	

Male	1.53 (1.09, 2.16)	0.02	1.83 (1.27, 2.63)	0.001

Race				

White	1		1	

Black	0.67 (0.44, 1.03)	0.06	0.76 (0.49, 1.17)	0.22

Other	1.23 (0.81, 1.88)	0.34	1.13 (0.73, 1.77)	0.58

Year of Injury (every year increment)	1.02 (0.97, 1.08)	0.44	1.02 (0.96, 1.08)	0.57

*Dependent variable is Falls < 3 meters vs. Other (without Falls > 3 meters)

**Dependent variable is Falls > 3 meters vs. Other (without Falls < 3 meters)

There were significant differences in proportions of injuries that resulted in abnormal CT scans, such that falls were more likely to be associated with abnormal CT scans than other types of injuries. Two-week mortality was significantly different by mechanism of injury (p < 0.0001) ranging from 18% due to injuries other than falls to 31% from falls < 3 meters (Table 4).

**Table 4:** Severity of injury and outcome by mechanism of injury

	Mechanism of Injury			
	Falls: < 3 meters N (%)	Falls: > 3 meters N (%)	Other N (%)	p-value

Day 1 GCS				

3 – 5	93 (47.5)	108 (44.4)	850 (51.3)	0.10

6 – 8	103 (52.5)	135 (55.6)	808 (48.7)	

Day 1 Hypotension	22 (11.0)	44 (17.4)	251 (14.8)	0.13

Day 1 Abnormal Pupil Status	37 (18.5)	47 (19.0)	356 (21.1)	0.56

Abnormal CT Scan	163 (89.6)	207 (88.5)	1301 (79.7)	<0.0001

Mortality	62 (30.7)	59 (24.8)	299 (17.5)	<0.0001

Outcome GCS				

3 – 8	47 (34.8)	64 (35.2)	446 (32.5)	0.67

9 – 15	88 (65.2)	118 (64.8)	927 (67.5)	

**Figure 1:**
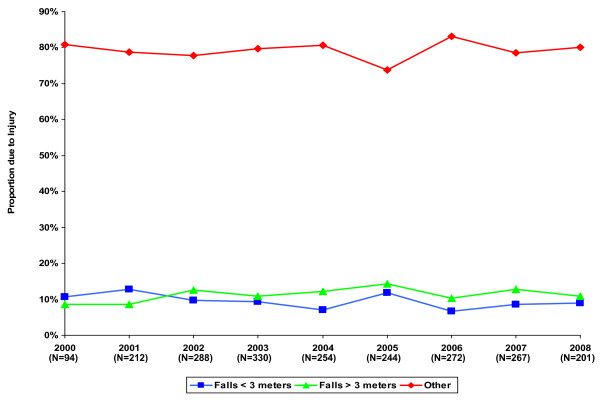
**Time Trends of Mechanism of Injury**.

Figure 1 displays the trends over time in the proportion of injuries due to falls from < 3 meters, falls from > 3 meters, and all other mechanisms of injury. The proportions due to these causes remain relatively stable during the course of the 9 years (2000–2008).

**Figure 2:**
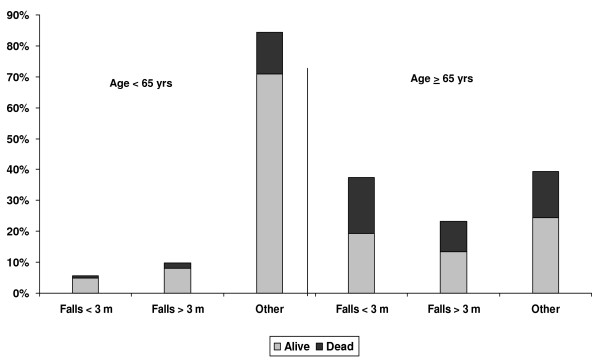
**Two-week Mortality by Mechanism of Injury and Age Group**.

Figure 2 displays the major mechanisms of injury by age group and mortality. Mortality was greater among patients 65 and older compared to those < 65 years within each mechanism of injury and resulted in especially high mortality among individuals experiencing low falls (48% mortality).

## Discussion

The results of this study are consistent with other reports documenting the greater frequency of motor vehicle crashes in younger individuals and the greater frequency of falls among the elderly [11,12]. Among those 65 and older, falls contributed to 61% of all injuries. Mortality was 31% and 25% due to falls from < 3 meters and > 3 meters for the entire study population, while among those ≥ 65 years reached 48% and 42%, respectively.

There are some limitations to this study. As noted above, the data are from 24 of 46 New York trauma centers representing 50% of the total trauma centers in the state. Since participation in the program is voluntary, data were not collected from the remaining trauma centers. However, since the participating trauma centers are distributed across the spectrum of urban and rural areas, we believe that the data recorded reflect a representative population.

The study does not account for TBI victims who die at the scene or in the emergency department because the database was designed to measure guideline compliance and inpatient mortality. It is the largest database of its kind to prospectively collect data to ascertain, describe, and follow severe TBI patients.

The database does not collect information on comorbidities and medications and other potentially important factors. In our experience, comorbidities and medications such as blood thinners appear to play an important role in the outcome from falls and traumatic brain injury especially in the elderly.

The significant relationship between increasing age and fall risk was especially striking. Even after controlling for patient demographic data, older age emerges as a strong independent factor affecting fall risk. Patients who are 65 years of age or older had a nearly 30-fold and almost nine-fold increased likelihood of low and high falls, respectively. These findings clearly demonstrate that in order to have an impact on mortality from severe traumatic brain injury, measures should be taken to prevent falls especially in the older population.

While other studies have documented the impact of falls on trauma among the elderly [10,11,13], they have often been outside the US [8,14] and have not focused on severe TBI. This study, using prospectively collected hospital-based data, underscores the recommendation that more effective preventive measures are needed to improve clinical outcomes of trauma among older people [8,15].

## Conclusion

The results of this study emphasize the importance of TBI as a major public health problem and demonstrate the need for tailored injury prevention programs according to the demographics of the population. For example, prevention programs for ages under 40 years need to focus on encouraging the use of seatbelts and helmets while efforts should focus on falls prevention and home safety among older people.

## Abbreviations

TBI: Traumatic brain injury; GCS: Glasgow Coma Scale; QI: Quality Improvement.

## Competing interests

The authors declare that they have no competing interests.

## Authors' contributions

LMG led the writing and directed all analyses of the article. QN constructed the study variables and conducted the data analyses. JG created the QI TBI database. RH and JG assisted in the interpretation of results and provided advice. All authors read and approved the final manuscript.

## References

[B1] Thurman D, Alverson C, Dunn K, Guerrero J, Sniezek J (1999). Traumatic brain injury in the United States: a public health perspective. J Head Trauma Rehabil.

[B2] Sousin DM, Sniezek JE, Waxweiller RJ (1995). Trends in death associated with traumatic brain injury, 1979 through 1992: success and failure. JAMA.

[B3] U.S. Department of Health and Human Services (1989). Interagency Head Injury Task Force Report.

[B4] (1995). Guidelines for the Management of Severe Head Injury.

[B5] Management and Prognosis of Severe Traumatic Brain Injury (2000). Part I Guidelines for the Management of Severe Traumatic Brain Injury.

[B6] (2007). Guidelines for the Management of Severe Traumatic Brain Injury,.

[B7] Masson F, Thicoipe M, Aye P, Mokni T, Senjean P, Schmitt V, Dessalles P-H, Cazaugade M, Labadens P, and the Aquitane Group for Severe Brain Injuries Study (2001). Epidemiology of severe brain injuries: a prospective population-based study. J Trauma.

[B8] Kennedy RL, Grant PT, Blackwell D (2001). Low-impact falls: demands on a system of trauma management, prediction of outcome, and influence of comorbidities. J Trauma.

[B9] Coronado VG, Thomas KE, Sattin RW, Johnson RL (2005). The CDC Traumatic Brain Injury Surveillance System: characteristics of persons aged 65 years and older hospitalized with a TBI. J Head Trauma Rehabil.

[B10] Helling TS, Watkins M, Evans LL, Nelson PW, Shook JW, Van Way CW (1999). Low falls: an underappreciated mechanism of injury. J Trauma.

[B11] Hannan EL, Waller CH, Farrell LS, Rosati C (2004). Elderly trauma inpatients in New York State 1994–1998. J Trauma.

[B12] Cooper A, Hannan EL, Bessey PQ, Farrell LS, Cayten CG, Mottley L (2000). An examination of the volume-mortality relationship for New York State trauma centers. J Trauma.

[B13] Hannan EL, Mendeloff J, Farrell LS, Cayten CG, Murphy JG (1995). Multivariate models for predicting survival of patients with trauma from low falls: the impact of gender and pre-existing conditions. J Trauma.

[B14] Lee KK, Seow WT, Ng I (2006). Demographical profiles of adult severe traumatic brain injury patients: implications for healthcare planning. Singapore Med J.

[B15] Richmond TS, Kauder D, Strumpf N, Meredith T (2002). Characteristics and outcomes of serious trauma injury in older adults. J Am Geriatr Soc.

